# ^18^F-PET-CT是Ⅰa期非小细胞肺癌术前筛查首选吗？

**DOI:** 10.3779/j.issn.1009-3419.2018.07.04

**Published:** 2018-07-20

**Authors:** 闯 何, 晶 袁, 玉潇 陈, 丽 杨, 良山 李, 廷源 李, 学全 黄

**Affiliations:** 400038 重庆，陆军军医大学（第三军医大学）第一附属医院放射科 Department of Radiology, Southwest Hospital, Third Military University (Army Medical University), Chongqing 400038, China

**Keywords:** 肺肿瘤, PET-CT, 无疾病进展期, Lung neoplasms, PET-CT, Progression-free survival

## Abstract

**背景与目的:**

低剂量螺旋计算机断层扫描（computed tomography, CT）筛查高度怀疑为肺癌的肺部阳性结节者，首选外科治疗。在实施外科治疗前如何选择全身筛查方式目前尚不清，本研究旨在探讨采用正电子发射型计算机断层显像（positron emission computed tomography, PET-CT）和常规影像（B-ultrasound/CT/MRI/ECT, BCME）对Ⅰa期非小细胞肺癌（non-small cell lung cancer, NSCLC）术前筛查后患者的无疾病进展期（progression-free survival, PFS）是否存在差异。

**方法:**

回顾性收集170例术前PET-CT筛查和130例BCME筛查的Ⅰa期NSCLC患者，将两组临床基本特征进行倾向值匹配分析（propensity score matching, PSM），两组分别有114例纳入研究。采用*Kaplan-Meier*生存曲线和*Cox*回归分析进行生存分析。

**结果:**

经PSM匹配后两组临床基本特征无显著差异。PET-CT组和BCME组的PFS分别为（44.9±27.2）个月、（44.1±33.1）个月，无显著差异（χ^2^=1.284, *P*=0.257）。PET-CT组术前筛查假阳性10例，BCME组8例，二者筛查假阳性率无显著差异（χ^2^=0.241, *P*=0.623），两种方式均能达到筛查目的，不是PFS的影响因素。

**结论:**

PET-CT和BCME均可用于Ⅰa期NSCLC根治术前筛查，可根据患者实际情况进行个体化选择。

## Abstract (en)

**Background and objective:**

The preferred therapy for patients with pulmonary nodules which highly suspected as lung cancer by low-dose spiral computed tomography (CT) is surgery, but the best screening method of whole body is not clear yet. The aim of this study is to investigate the differences in the progression-free survival (PFS) of patients with Ⅰa stage non-small cell lung cancer after screening of positron emission computed tomography (PET)-CT and conventional imaging (B-ultrasound/CT/MRI/ECT, BCME).

**Methods:**

A total of 300 cases of Ⅰa stage non-small cell lung cancer were collected, of which 170 cases were performed PET-CT and 130 cases were performed BCME before operation. The basic characteristics of the two groups were analyzed by propensity score matching (PSM), and 114 cases of each group were included in the study. The survival analysis was carried out by the *Kaplan-Meier* survival curve and the *Cox* regression analysis.

**Results:**

There was no significant difference between each group analyzed by PSM. The PFS of PET-CT and BCME were (44.9±27.2) months and (44.1±33.1) months (χ^2^=1.284, *P*=0.257). Both of the method ssucceed in screening. It is not the PFS influence factors. The false positive of PET-CT and BCME were 10 cases and 8 cases (χ^2^=0.241, *P*=0.623).

**Conclusion:**

Both PET-CT and BCME can be used as a screening method for Ⅰa stage non-small cell lung cancer according to individualized choice of patients.

低剂量螺旋计算机断层扫描（computed tomography, CT）在肺癌高危人群进行肺癌筛查能降低20%的肺癌死亡^[1]^，低剂量螺旋CT筛查高度怀疑为肺癌的肺部阳性结节者，需进行多学科讨论，对于适合于外科手术治疗者，一定首选外科治疗^[2]^。正电子发射型计算机断层显像（positron emission computed tomography, PET）-CT在非小细胞肺癌（non-small cell lung cancer, NSCLC）分期中的作用毋庸置疑，但是从我国卫生经济学考虑，2017版中国临床肿瘤学会（Chinese Society of Clinical Oncology, CSCO）指南将PET/CT作为可选择策略的2A类证据推荐。高危人群低剂量CT筛查怀疑肺癌的阳性结节，评估可手术治疗者，术前全身筛查方式目前未有更多研究。本研究主要目的是回顾性分析我院10年内Ⅰa期（T≤3 cm）NSCLC术前筛查方式选择，了解采用PET-CT和BCME术前筛查后患者的无疾病进展期（progression-free survival, PFS）情况。

## 1 资料与方法

### 1.1 临床资料

选择陆军军医大学第一附属医院2007年1月-2017年12月期间行肺癌根治性手术切除的Ⅰa期NSCLC患者366例，排除多源癌、术后短期内并发症死亡、磨玻璃结节患者。依据国际抗癌联盟（Union for International Cancer Control, UICC）肺癌肿瘤-淋巴结-转移（tumor-node-metastasis, TNM）分期第8次修订版，Ⅰ期分为Ⅰa期和Ⅰb期，其中Ⅰa期又分为Ⅰa1期、Ⅰa2期、Ⅰa3期。本研究将2007年-2016年资料按TNM分期第8次修订版进行再分期。最终300例患者纳入本研究，男性169例，女性131例，平均年龄（56.5±9.35）岁（34岁-77岁），在Ⅰa期NSCLC中，T1aN0M0（≤1 cm）28例，T1bN0M0（1 cm-2 cm）169例，T1cN0M0（2 cm-3 cm）103例，术后病理诊断包括腺癌243例，鳞癌46例，其他类型11例。170例术前PET-CT筛查，130例术前BCME筛查，BCME术前筛查方法包括腹部B超、胸部CT、颅脑磁共振成像（magnetic resonance imaging, MRI）或CT、全身骨扫描（emission computed tomography, ECT）。将临床基本特征按1:1 PSM分析后，两组分别纳入114例患者进入研究（表 1），男性138例，女性90例，平均年龄（56.8±9.4）岁（35岁-77岁）。生存时间从手术日起，截止随访时间为2017年12月31日，平均随访时间（44.7±30.2）个月（4个月-129个月）。

**1:** 患者临床资料 Clinical characteristics of patients after PSM

Clinical factors	PET-CT	BCME	*P*
Gender			0.416
Male	72	66	
Female	42	48	
Age (yr)			0.686
≥60	45	48	
< 60	69	66	
Smoker			0.427
Yes	61	55	
No	53	59	
T stage			0.861
T1a	8	10	
T1b	73	70	
T1c	33	34	
Location (lung)			0.110
Left	57	45	
Right	57	69	
Pathology			0.745
AD	91	89	
N-Ad	23	25	
Chemotherapy			0.232
Yes	65	56	
No	49	58	
AD: adenocarcinoma; N-Ad: non-adenocarcinoma; PSM: propensity score matching; PET: positron emission computed tomography; CT: computed tomography; BCME: B-ultrasound/CT/MRI/ECT; MRI: magnetic resonance imaging; ECT: emission computed tomography.			

### 1.2 研究终点

主要研究终点是PFS，次要观察指标为筛查假阳性率和总生存期（overall survival, OS）。无疾病进展期定义为：手术日至首次确诊远处转移或未发生转移的末次随访时间。假阳性定义为：PET-CT或BCME既往影像诊断报告怀疑转移，在随访过程中排除为转移灶。总生存期定义为手术日至末次随访或任何原因死亡时间。

### 1.3 统计学分析

采用SPSS Statistics 24进行统计学分析。两组资料按1:1倾向性评分匹配，“卡钳值”设为0.02。正态数据用（Mean±SD）表示，率比较采用卡方检验，生存分析采用*Kaplan-Meier*生存曲线，组间采用*Log-rank*（*Mantel-Cox*）比较。采用单因素*Cox*回归分析以及多因素*Cox*回归（Forward:LR）生存分析，以*P* < 0.05为差异有统计学意义。

## 2 结果

### 2.1 临床特点

本研究经PSM匹配分析后，共228例Ⅰa期NSCLC纳入研究，两组基本临床特征无显著差异。至末次随访时PET-CT组，复发转移14例（12.2%），BCME组，复发转移19例（16.6%），两组复发转移率无显著差异（χ^2^=0.886, *P*=0.347），复发转移部位主要发生在肺、纵隔、骨骼和颅脑。至末次随访时间，26例患者死亡，其中25例发生肿瘤相关性死亡（11.0%），1例死于主动脉夹层破裂。

### 2.2 筛查假阳性率

PET-CT组出现假阳性10例，BCME组出现假阳性为8例，两组筛查的假阳率无显著差异（χ^2^=0.241, *P*=0.623）。

### 2.3 生存分析

经*Kaplan-Meier*生存分析显示。PSM匹配分析后，PET-CT组和BCME组的PFS分别为（44.9±27.2）个月、（44.1±33.1）个月，两组PFS无显著差异（χ^2^=1.284, *P*=0.257）（图 1）。OS分别为（47.1±27.0）个月、（46.0±33.1）个月。通过*Cox*单因素分析发现肿瘤分期是PFS的危险因素（*P*=0.044）。*Cox*多因素分析发现肿瘤分期是PFS的风险因素（*P*=0.018），且非腺癌相较于腺癌术后更容易复发转移（*P*=0.036），而选择筛查方式不是PFS的影响因素（表 2）。

**1:**
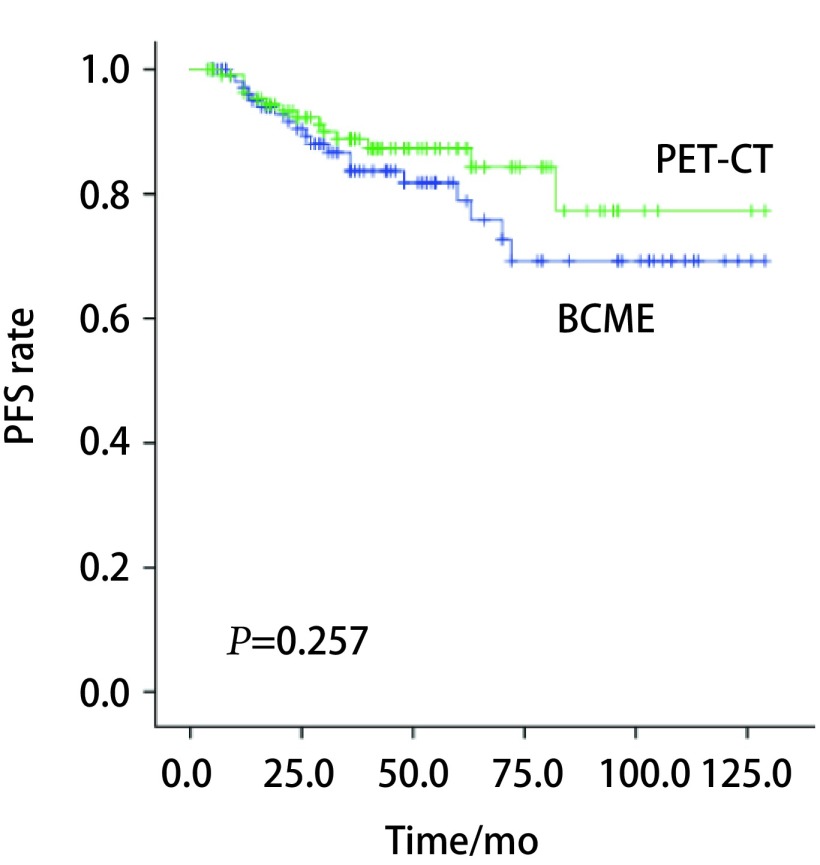
两组PFS情况。PET-CT组和BCME组比较，两组PFS无显著差异（*χ*^2^=1.284, *P*=0.257）。 PFS rates in patients with Ⅰa stage non-small cell lung cancer. PFS was no significant difference in PET-CT group compared with BCME group (*χ*^2^=1.284, *P*=0.257).

**2:** PFS的预测因素 Univariable and multivariable analyses of predictors of PFS after PSM

Clinical factors	Univariable analysis of PFS							Multivariable analysis of PFS					
	B	SE	Wald	*P*	HR	95%CI		B	SE	Wald	*P*	HR	95%CI
Gender (female *vs* male)	-0.045	0.363	0.015	0.902	1.046	0.514-2.128							
Age (< 60 *vs* ≥60)	-0.295	0.364	0.655	0.418	0.745	0.365-1.521							
Smoke (Yes *vs* No)	-0.148	0.353	0.176	0.675	1.16	0.581-2.315							
Pathology (Ad *vs* N-Ad)	-0.969	0.536	3.268	0.071	0.379	0.133-1.085		-1.133	0.541	4.391	0.036	0.322	0.112-0.929
T stage	0.636	0.315	4.073	0.044	1.889	1.019-3.503		0.761	0.321	5.62	0.018	2.141	1.141-4.018
Location (left *vs* right lobe)	-0.526	0.350	2.255	0.133	0.591	0.298-1.174							
Chemotherapy	-0.470	0.367	1.642	0.200	0.625	0.304-1.283							
PET-CT or BCME	-0.396	0.353	1.262	0.261	0.673	0.337-1.343							

## 3 讨论

早期肺癌可以治愈，但是2/3的患者发现时已为晚期^[3]^。随着低剂量CT筛查手段在高危人群中的应用，越来越多的早期肺癌结节被发现，降低了肺癌的死亡率^[1]^。手术切除是NSCLC治愈主要手段之一，Ⅰa期NSCLC的5年存活率 > 75%^[4]^，在不增加并发症发生率的情况下，每个病人在根治性手术切除时都要行纵隔淋巴结取样^[5]^。^18^F-FDG PET-CT对NSCLC患者的筛查有助于临床医生选择，且患者能从手术中获益，但对淋巴结转移的鉴别具有较高的假阳性率^[6]^，本研究中两组术前筛查方式均出现一定的假阳性率，但两组假阳性率的发生无显著差异，在临床随访过程中均已排除。2018版美国国立综合癌症网络（National Comprehensive Cancer Network, NCCN）指南建议 > 8 mm的肺部结节推荐PET-CT诊断或组织活检诊断^[7]^，随着目前经皮穿刺活检、支气管内镜器械和技术的发展，术前病理诊断比较容易，但是筛查指南建议由于肿瘤原因、患者心肺功能异常不能耐受外科手术治疗，或者患者本人不愿意接受外科手术治疗者方可实施穿刺活检^[2]^，笔者建议在明确病理诊断后有条件者行PET-CT筛查。对于在高危人群中低剂量CT筛查出怀疑肺癌的阳性结节（Ⅰa期），PET-CT筛查在这部分人群中获益多少仍不清楚。

本研究对选用两种术前筛查方式患者的PFS作对比，至截访日，本研究组死亡26例，其中1例非肿瘤性死亡，因结局事件数较少可能会导致数据过度拟合，故本研究未将OS纳入分析。本组出现疾病进展33例，平均出现时间（30.3±20.8）个月，基本可以排除因术前筛查漏诊导致PFS的缩短，经多因素分析也证明，选择PET-CT术前筛查并不一定是PFS的影响因素。T分期较早也可能存在远处转移，常规BCME检查可能会出现漏诊，PET-CT对于淋巴结转移和胸腔外转移有更好的诊断效能^[8]^，若病灶较小，因肝脏、大脑皮髓质交接区代谢较高，易出现假阴性结果，相较于常规筛查略显不足。上述两种术前筛查方式都可能存在漏诊，导致筛查的不准确，本组数据显示筛查漏诊的几率不高，PFS未受筛查方式影响。但是两种筛查均存在一定假阳性率，在临床中需要谨慎对待此类病例。

综上所述，通过本组回顾性数据提示，当发现肺部可疑Ⅰa期肺癌时，选择PET-CT和BCME进行术前全身筛查均能达到临床需求，筛查后患者的PFS无显著差异，在临床抉择过程中需进行个体化选择。
